# Adolescent non-suicidal self-injury scale – short form: revision and evaluation of its reliability and validity

**DOI:** 10.3389/fpsyt.2026.1911843

**Published:** 2026-09-28

**Authors:** Ao Li, Xiaoxiao Song, Xiaoyan Wang, Quanwei Zheng, Xu Zhang, Mengxin Sun, Shaobo Lyu

**Affiliations:** 1 Hebei Key Laboratory of Mental Health and Brain Science, School of Psychology and Mental Health, North China University of Science and Technology, Tangshan, Hebei, China; 2 Library, North China University of Science and Technology, Tangshan, Hebei, China; 3 Guokai Experimental School, Tangshan, Hebei, China

**Keywords:** adolescents, genetic algorithm, non-suicidal self-injurious behavior, rapid screening, scale revision

## Abstract

**Objective:**

Non-suicidal self-injury (NSSI) is a clinically significant concern among adolescents, and its accurate yet efficient assessment is essential for research, early identification, and intervention. Existing Chinese instruments—most notably the 19-item Adolescent Self-Harm Questionnaire (ASHS)—are relatively lengthy and ill-suited to rapid, large-scale screening. The present study had two aims: (a) to develop a short form of the ASHS, the Adolescent Non-Suicidal Self-Injury Scale–Short Form (ANSSI-SF), by integrating classical test theory (CTT) item analysis with genetic algorithm (GA) cross-validation, and (b) to evaluate its reliability, validity, and screening utility relative to the parent scale.

**Methods:**

Using a convenience sample of 1,090 primary and secondary school students, seven items—one for each of seven behavioral categories derived from a functional–typological framework of self-harm—were selected on the basis of CTT item statistics and GA cross-validation. The scale was evaluated through item analysis, internal-consistency and split-half reliability, exploratory and confirmatory factor analysis (EFA/CFA), correlations with theoretically related constructs, known-groups comparisons (gender; smartphone addiction), and consistency and screening-accuracy analyses against the full 19-item ASHS.

**Results:**

It indicated that all items demonstrated strong correlations with the total score, and both internal consistency and split-half reliability reached satisfactory levels.A unidimensional factor structure also exhibited good model fit. Additionally, scale scores were significantly positively correlated with related psychological indicators such as depression, anxiety, and sleep problems, and effectively differentiated differences in self-injurious behavior across gender and smartphone addiction groups.GA cross-validation confirmed that the selected items balanced parsimony with full coverage of all seven behavioral categories.

**Discussion:**

These findings indicate that the ANSSI-SF is a brief, reliable, and valid instrument that preserves the content breadth of the parent ASHS while substantially reducing administration burden. It is well suited to rapid, large-scale screening and early identification of at-risk adolescents, facilitating timely referral and prevention. Limitations include convenience sampling from a single region and the absence of test–retest reliability; future research should validate the scale in clinical and diverse cultural samples and examine its predictive validity.

## 1 Introduction

Non-suicidal self-injury (NSSI) refers to the intentional, repetitive infliction of injury on one’s own body tissue without suicidal intent, carried out in a non-lethal or low-lethality manner (1, 2).It is widely acknowledged that NSSI behaviors are positively associated with negative emotional states such as depression, anxiety, experiential avoidance, sleep disturbances, and diminished levels of positive cognition (3–7).There are notable individual differences in the risk of self-injurious behaviors; while extreme stress may elicit self-injurious impulses in many individuals, those with specific psychological vulnerabilities tend to develop this behavioral pattern more readily and more frequently (8, 9).

Valid assessment of NSSI is fundamental to rigorous research and effective clinical intervention. Internationally, several instruments are available for this purpose. The Inventory of Statements About Self-Injury (ISAS; 10) is among the most widely used, as it assesses both the functions (13 functional domains) and the specific methods (12 behavioral categories) of NSSI and exhibits robust psychometric properties; however, its functional section is lengthy and oriented toward understanding the motives underlying self-injury rather than toward rapid behavioral screening. Within China, Zheng (11) developed the first adolescent NSSI questionnaire on the basis of in-depth interviews with adolescents. Although this instrument served as an important reference for subsequent domestic research, it has been criticized for limited item clarity and incomplete coverage of NSSI types. To address these limitations, Feng (12) systematically revised the instrument and developed the Adolescent Self-Harm Questionnaire (ASHS), which includes 19 items assessing both the frequency and severity of self-injurious behaviors. This scale demonstrates sound psychometric properties and offers a practical indicator for quantifying the severity of NSSI by integrating frequency and severity scores, leading to its broader use in related studies within China. In the present study, the ASHS was selected as the parent instrument for three reasons: (a) it represents the de facto standard for adolescent NSSI assessment in China; (b) its item content directly covers the behavioral forms of NSSI observed in Chinese adolescents; and (c) its 19 items provide sufficient breadth to support abbreviation while preserving content coverage.

However, the increasing demand for epidemiological surveys of NSSI and for large-scale mental-health screening in school settings may render the length of the full ASHS a practical limitation. Lengthy questionnaires tend to increase respondent fatigue and resistance, particularly for sensitive topics such as self-injury, which may compromise data authenticity and reduce completion rates. In contexts in which time and resources are constrained and rapid identification of high-risk adolescents is required, a concise and effective screening instrument is of greater practical value. To date, however, no simplified version of the ASHS is available in China, and empirical short-form development for Chinese adolescent NSSI instruments remains lacking.

In view of this, the present study had two aims: (1) to develop a short form of the ASHS—the Adolescent Non-Suicidal Self-Injury Scale–Short Form (ANSSI-SF)—guided by classical test theory (CTT; 13), and (2) to evaluate the reliability, validity, and screening utility of this short form relative to the parent scale. As a methodological complement, the item-selection solution was cross-validated using a genetic algorithm (GA; 14), a heuristic optimization algorithm inspired by Darwinian evolutionary principles. Through selection, crossover, and mutation, the GA explores the high-dimensional subset search space and converges on item subsets that maximize the variance of the original scale explained while minimizing item count (15, 16).Because GA searches are probabilistic, solutions were stabilized across repeated independent runs using majority-based item selection (17).Although GA-based scale abbreviation has been applied in other domains (e.g., the Multidimensional Experiential Avoidance Questionnaire; 16), to the best of our knowledge this is the first application of a GA to abbreviate the ASHS or any Chinese adolescent NSSI instrument.

To ensure that brevity does not compromise content breadth, item selection was organized around the functional and typological framework of self-harm behaviors (18), which distinguishes the core behavioral types of self-injury. On the basis of this framework and the item content of the ASHS, seven core behavioral categories were defined *a priori*: cutting, hitting, scratching, piercing/inserting objects, burning/scalding, tattooing/marking, and other forms. Retaining one item per category ensures that the short form captures the full range of adolescent self-injurious behaviors rather than over-representing the most frequent forms. From a clinical perspective, the 7-item scale is easy to incorporate into routine school mental health screening batteries, repeated monitoring, and large-scale epidemiological surveys. This brevity is expected to reduce participant fatigue and refusal to answer questions on sensitive topics, improve data quality, and—by enabling rapid, repeated assessments—facilitate the early identification of at-risk adolescents and their timely referral to specialized services.

## 2 Methods

### 2.1 Subjects

A convenience sampling strategy was adopted. Participants were recruited from Grades 4–9 in two schools in Tangshan, China (Lingang School and Guokai Experimental School), and all students in the selected classes were invited to participate; all participants provided written parental/guardian informed consent and personal assent. Of the 1,203 questionnaires distributed, 1,090 valid questionnaires were returned (missing rate = 9.39%).The final sample comprised 1,090 students (580 boys, 510 girls) aged 9–15 years (M = 11.43, SD = 2.08), deliberately including late-primary-school students because NSSI onset frequently occurs in early adolescence. Students with severe reading or cognitive difficulties or with excessive missing data were excluded. All participants completed the 19-item ASHS, all criterion questionnaires, and the Smartphone Addiction Scale (SAS).For the remaining questionnaires, missing values were rare and were handled by listwise deletion, and no outliers were removed because NSSI scores are legitimately highly skewed in general-population samples.

The full sample was randomly divided into two equal halves (n = 545 each) for EFA (subsample A) and CFA (subsample B), providing internal cross-validation of the factor structure; item analysis and reliability estimation used the full sample. In addition, 218 participants with the highest and lowest 10% of SAS scores (n = 109 per group) were selected for known-groups comparisons. Because NSSI scores are highly skewed, this extreme-groups design was used; smartphone addiction was chosen as the grouping variable on the basis of replicated evidence linking problematic smartphone use to NSSI in Chinese adolescents (19–21), and the analysis serves as supplementary known-groups evidence rather than primary criterion validation. The full sample exceeded the recommended 10 participants per item and 300 participants for factor analysis (22).

### 2.2 Questionnaire development

The ANSSI-SF was developed directly in Chinese from the Chinese-language ASHS (12), with no translation involved. The parent ASHS comprises 19 items assessing the frequency and severity of self-injurious behaviors and is summarized by a single total score (the sum of frequency × severity products across items).To ensure that the shortened scale retained the core behavioral types of adolescent self-injury, item selection was organized around the functional and typological framework of self-harm behaviors (18), which distinguishes the core behavioral types of self-injury. On the basis of this framework and the item content of the ASHS, seven core behavioral categories were defined: cutting, hitting, scratching, piercing/inserting objects, burning/scalding, tattooing/marking, and other forms. Within each category, item retention was guided by classical item-analysis statistics (item–total correlations, corrected item–total correlations, and the effect of item deletion on Cronbach’s α); items were retained if their item–total correlation was ≥ 0.40 and their corrected item–total correlation was ≥ 0.30 and if deletion did not substantially increase internal consistency. Rather than simply retaining individual original items, the final item within each category was formed by refining the candidate items that met these criteria and merging closely related content into a single representative item, so that each item preserved the core behavioral meaning of its category and the seven-item scale represented every core behavioral category. Scoring followed the original ASHS: each item is scored as the product of frequency (0–3) and severity (1–5), and the total score is the sum of the item scores. Item selection was conducted on the full sample (n = 1,090).

To cross-validate the item-selection solution with an independent method, a genetic algorithm was implemented in R using the GAabbreviate package (16), which is built on the GA package (14). The GA cross-validation was performed on the full sample (n = 1,090).The fitness (objective) function minimized cost = (1 − R²) + c × k, where R² is the variance of the original ASHS total score explained by the selected items, k is the number of selected items, and c = 0.05 is a parsimony penalty per item. Following the GA package defaults, the algorithm used a population size of 50, crossover and mutation rates of 0.8 and 0.1, respectively, a maximum of 200 generations, and linear-rank selection; convergence was defined as no improvement in fitness for 25 consecutive generations. Because GA searches are probabilistic, 25 independent runs were performed, and items were selected on the basis of the frequency with which they were included across runs (**Figure 1** shows a representative run). Because an unconstrained GA over-represents the most frequent behaviors, a two-stage procedure was used: after the most efficient items were selected in Stage 1, they were removed and a second GA was run on the remaining pool to ensure coverage of all seven categories. The choice of seven items—one per category—was supported empirically: a six-item solution captured approximately 90% of the parent-scale variance but left one category unrepresented, whereas seven items achieved full coverage at minimal length. This hybrid approach was adopted because purely manual item selection is subjective, and purely statistical criteria alone may not ensure broad content coverage; the GA thus provides an objective, reproducible search that corroborates or challenges the CTT-driven item set.

**Figure 1:**
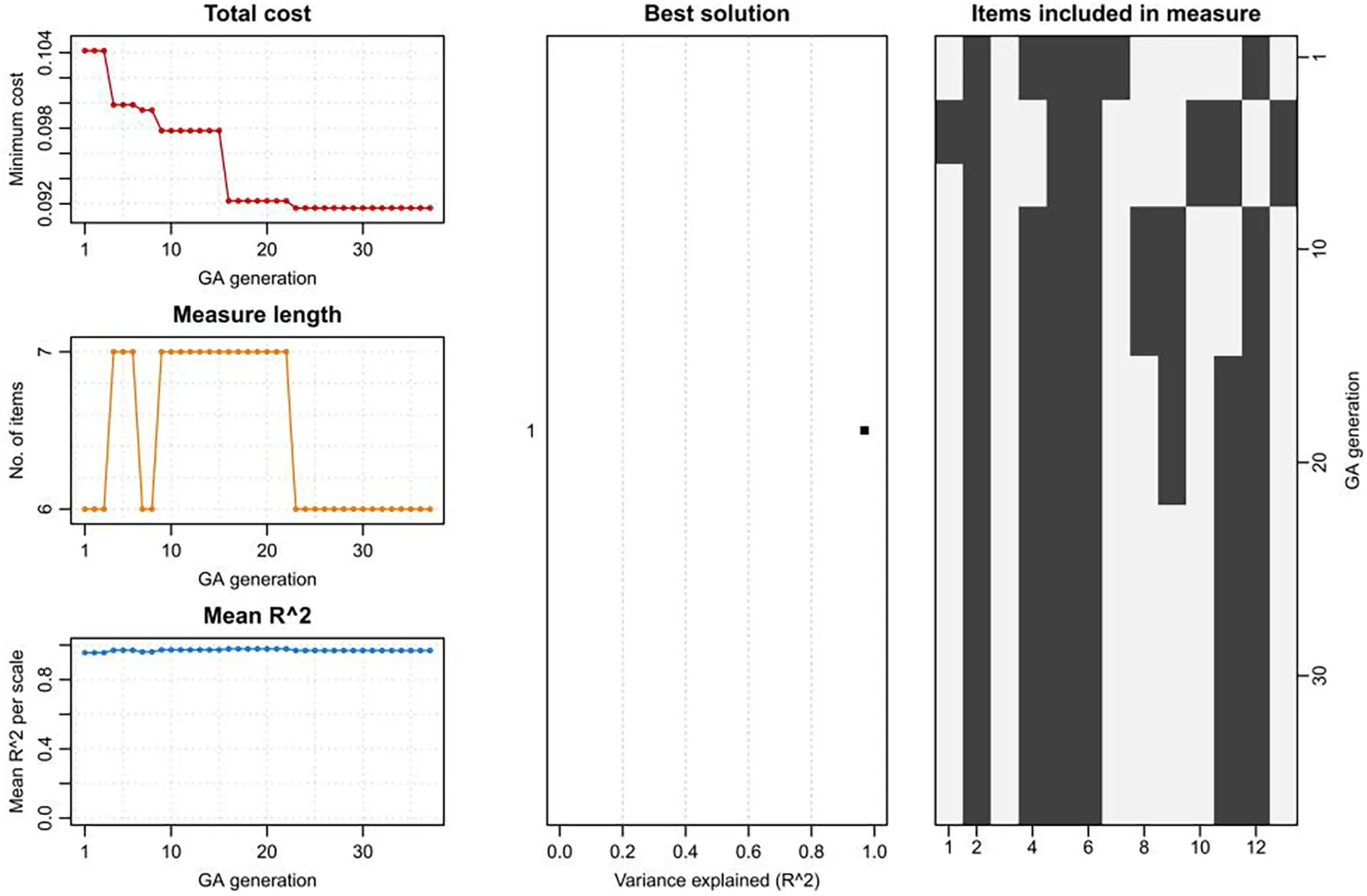
Results of genetic algorithm screening for scale items (a typical outcome from one of 25 independent runs).Subfigures: **(a)** minimum cost across generations; **(b)** number of selected items across generations; **(c)** average explained variance (R²) across generations; **(d)** variance explained by the final selected item combination for the single dimension; **(e)** heatmap of item selection status across generations.

### 2.3 Criterion tools

Criterion-related validity was evaluated using the following measures: depression (PHQ-9), anxiety (GAD-7), experiential avoidance (AAQ-II), sleep problems (PSQI), mindfulness (CAMM), and smartphone addiction (SAS).On the basis of the literature, ANSSI-SF scores were expected to correlate positively with depression, anxiety, experiential avoidance, sleep problems, and the full ASHS, and negatively with mindfulness, operationalized here as a positive correlation with CAMM total scores, because higher CAMM scores in this study indicate lower mindfulness. All questionnaires were self-administered in class under researcher supervision, in a fixed order (ASHS first), taking approximately 30 minutes.

#### 2.3.1 Patient health questionnaire-9 (PHQ-9)

The scale (23) is used to assess the severity of depressive symptoms in the past two weeks and consists of nine entries, including how much time was spent suffering from nine problems including lack of interest and low mood. A 4-point scale was used for scoring (0, “Never”; 3, “Almost every day”). This scale demonstrates good reliability and validity in adolescent populations. In this study, the overall Cronbach’s α coefficient for the PHQ-9 was 0.90.

#### 2.3.2 Generalized anxiety disorder-7 (GAD-7)

This scale (24) assesses the severity of generalized anxiety symptoms over the past two weeks. It consists of seven items evaluating symptoms such as nervousness, anxiety, and uncontrollable worry. A 4-point scoring scale was used (0, “Never”; 3, “Almost every day”). This scale has demonstrated good reliability and validity in adolescent populations. In this study, the overall Cronbach’s α coefficient for the GAD-7 was 0.93.

#### 2.3.3 Acceptance and action questionnaire-II (AAQ-II)

This study employed the Chinese version of the scale (25), which retained the original single-dimensional structure comprising seven items for assessing experiential avoidance. Scoring utilized a 7-point scale (1, “never”; 7, “always”). The scale demonstrated good reliability and validity in adolescent populations. In this study, the AAQ-II achieved an overall Cronbach’s α coefficient of 0.93.

#### 2.3.4 Pittsburgh sleep quality index

The PSQI was developed by Buysse et al. (1989) (26); its Chinese version was developed and validated by Liu Xianchen et al. (1996) (27). This instrument assesses subjective sleep quality over the past month and comprises 19 items across seven dimensions, including subjective sleep quality and sleep latency. A 4-point scoring system was used (0, “no difficulty”; 3, “severe difficulty”).This scale has demonstrated good reliability and validity in adolescent populations. In this study, the overall Cronbach’s α coefficient for the PSQI was 0.80.

#### 2.3.5 Child and adolescent mindfulness measure

The Chinese version of the CAMM, developed by Greco et al. (28) and translated by Liu Xiaofeng et al. (29), has been validated for reliability and validity among Chinese adolescents. It measures children and adolescents’ ability to notice and accept present experiences. The Chinese CAMM features a single-dimensional structure comprising 10 items, scored on a five-point scale (1 = “Never,” 5 = “Always”). In the present sample, CAMM item scores were summed without reverse coding, such that higher total scores indicate lower mindfulness; the Cronbach’s α coefficient for this scale was 0.90.

#### 2.3.6 Smartphone addiction scale (SAS)

Smartphone addiction was assessed with the Chinese version of the SAS (30), which demonstrated good internal consistency in the present sample. SAS scores were used to select the extreme groups (top and bottom 10%) for the known-groups analysis. In this study, the Cronbach’s α coefficient for this scale was 0.93.

### 2.4 Statistical methods

Statistical analyses were conducted with SPSS 25.0, Amos 24.0, and R (GA package; 14).SPSS 25.0 was used for item analysis, exploratory factor analysis (EFA; principal-component extraction, no rotation because a single factor was extracted), and internal-consistency reliability of the initial scale, and for descriptive statistics and correlation analyses of the criterion measures for the simplified scale. Amos 24.0 was used for the confirmatory factor analysis (CFA; maximum-likelihood estimation) of the simplified scale to verify its construct validity, with model fit evaluated using χ²/df ≤ 3, CFI and TLI ≥ 0.95, and RMSEA and RMR ≤ 0.08; R (GA package; 14) was used for the genetic algorithm cross-validation. Reliability was assessed with Cronbach’s α and Spearman–Brown split-half reliability. Criterion-related validity was examined with Pearson correlations and known-groups t tests, and screening utility with a ROC analysis of the ANSSI-SF total score against full-ASHS-based NSSI status (AUC, sensitivity, specificity, Youden-index-optimal cutoff).Significance was set at p < 0.05 (two-tailed); given the number of correlations, p < 0.01 coefficients were treated as the primary validity evidence.

### 2.5 Ethical approval

This study was approved by the Ethics Committee of North China University of Science and Technology (Approval No.2024088; Issue Date: 20240305).Informed assent was obtained from all youth participants, and written informed consent was obtained from all participants’ parents or guardians. Participation was voluntary and anonymous; data were stored securely and used for research purposes only.

## 3 Results

### 3.1 Item analysis

Item analysis showed that item scores on the ANSSI-SF correlated with the total score between 0.54 and 0.87 (all p < 0.01; **Table 1**).Except for Item 6 (r = 0.54), all item–total correlations exceeded 0.70, indicating that most items showed good consistency with the overall scale and satisfactory discrimination; the correlation for Item 6, although lower, remained acceptable. Item-level descriptive statistics are presented in **Table 2**.

**Table 1:** Correlation coefficients between items and total score, and factor loadings for the adolescent non-suicidal self-injury scale–short form (ANSSI-SF).

Item	Correlation coefficient r between item and total score (n=1090)	Factor loadings (n=545)
1. Intentionally cutting oneself	0.838***	0.845
2. Intentionally hitting oneself causing bruises	0.873***	0.906
3. Intentionally burning/scalding oneself	0.705***	0.732
4. Intentionally tattooing or marking one’s body (excluding cosmetic tattoos)	0.841***	0.853
5. Intentionally scratching one’s skin until bleeding	0.829***	0.782
6. Intentionally inserting objects into skin or under fingernails	0.544***	0.505
7. Other forms of intentional self-harm	0.835***	0.851
Feature Root		4.389
Explained Variance		62.70%

***P<0.001.

**Table 2:** Item-level descriptive statistics for the ANSSI-SF (n = 1,090).

Item	M	SD	Endorsement rate (%)	Skewness
1. Intentionally cutting oneself	0.42	1.70	9.6	5.60
2. Intentionally hitting oneself causing bruises	0.22	1.32	5.0	8.53
3. Intentionally burning/scalding oneself	0.16	1.02	4.0	8.93
4. Intentionally tattooing or marking one’s body (excluding cosmetic tattoos)	0.29	1.56	6.3	7.19
5. Intentionally scratching one’s skin until bleeding	0.26	1.36	6.6	7.66
6. Intentionally inserting objects into skin or under fingernails	0.17	0.91	4.8	6.82
7. Other forms of intentional self-harm	0.29	1.52	6.6	7.16

M, mean; SD, standard deviation; endorsement rate, percentage of participants with a non-zero item score.

### 3.2 Reliability analysis

Internal consistency and split-half reliability were computed to evaluate the reliability of the scale. Split-half reliability was 0.92 and Cronbach’s α was 0.895. As shown in **Table 3**, all corrected item–total correlations exceeded 0.60 except Item 6 (0.45), which remained above the acceptable threshold of 0.30; deleting Item 6 would increase α only marginally (to 0.904).Because Item 6 measures a distinct, clinically meaningful type of NSSI (object insertion) not covered by other items, it was retained on content-validity grounds.

**Table 3:** ANSSI-SF item–total statistics.

Item	Correlation between modified items and total	Cronbach’s alpha after item deletion
1. Intentionally cutting oneself	0.746	0.876
2. Intentionally hitting oneself causing bruises	0.819	0.865
3. Intentionally burning/scalding oneself	0.626	0.889
4. Intentionally tattooing or marking one’s body (excluding cosmetic tattoos)	0.761	0.872
5. Intentionally scratching one’s skin until bleeding	0.757	0.872
6. Intentionally inserting objects into skin or under fingernails	0.450	0.904
7. Other forms of intentional self-harm	0.755	0.872

### 3.3 Factor analysis

EFA on subsample A (n = 545) yielded a KMO of 0.85; both the eigenvalue-greater-than-one rule (eigenvalue = 4.389) and the scree plot supported a single factor accounting for 62.70% of the total variance, and parallel analysis based on 1,000 random permutations (31) corroborated the one-factor solution: the first observed eigenvalue (4.389) exceeded the 95th-percentile random eigenvalue (1.163), whereas the second observed eigenvalue (0.936) fell below its random counterpart (1.095).Factor loadings ranged from 0.51 (Item 6) to 0.91 (Item 2), all above the 0.40 retention threshold (**Table 1**).

CFA on subsample B (n = 545) confirmed the single-factor model with good fit: χ²/df = 3.01, CFI = 0.996, TLI = 0.981, RMSEA = 0.061, RMR = 0.020. All standardized loadings were ≥ 0.70 (p < 0.01) except Item 3 (0.63) and Item 6 (0.52), which exceeded the common inclusion threshold (32, 33; **Figure 2**). Measurement invariance was not examined and is noted as a limitation.

**Figure 2:**
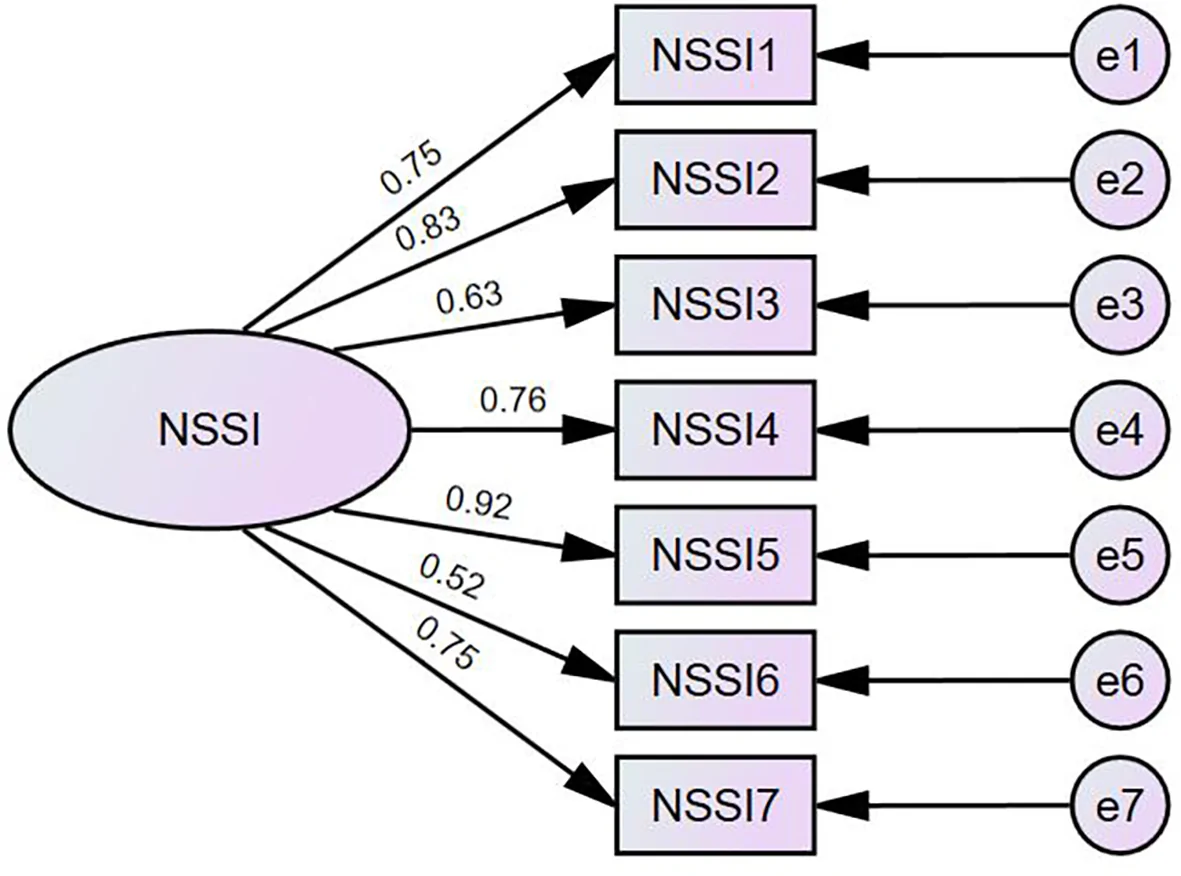
Confirmatory factor loading plot for the seven-item single-factor model of the Adolescent Non-Suicidal Self-Injury Scale–Short Form (ANSSI-SF).All factor loadings are standardized and significant at p < 0.01, except item 6 (loading = 0.52, p < 0.05).

### 3.4 Validity analysis

**Table 4** presents descriptive statistics and correlations. ANSSI-SF scores were strongly correlated with the full 19-item ASHS (r = 0.889, p < 0.001); because the two forms share item content, this high coefficient is interpreted as an index of consistency with the parent scale rather than independent criterion validity.

**Table 4:** Descriptive statistics of the outcome measures and correlations with the ANSSI-SF.

Measure	Mean (M)	Standard deviation (SD)	ANSSI-SF total score (r)
Full ASHS (19 items; parent scale)	6.87	23.61	0.889***
PHQ-9	2.60	3.51	0.471***
GAD-7	1.75	2.97	0.524***
AAQ-II	14.32	8.35	0.432***
PSQI	1.94	2.29	0.399***
CAMM	9.57	8.54	0.335***

***p < 0.001.

Consistent with hypotheses, ANSSI-SF scores correlated positively with depression (PHQ-9, r = 0.471), anxiety (GAD-7, r = 0.524), experiential avoidance (AAQ-II, r = 0.432), and sleep problems (PSQI, r = 0.399; all p < 0.001), and positively with CAMM scores (r = 0.335, p < 0.001).Because higher CAMM scores in this study indicate lower mindfulness, this positive correlation reflects the expected inverse association between mindfulness and NSSI.

The criterion measures—especially the full ASHS (M = 6.87, SD = 23.61)—showed low means and large SDs, reflecting the highly skewed, low-base-rate nature of NSSI combined with frequency × severity product scoring; this pattern is typical of NSSI instruments in general-population samples. The ANSSI-SF total score was similarly highly skewed (M = 1.80, SD = 7.50, skewness = 6.89, kurtosis = 58.52), with 16.8% of participants obtaining a non-zero total score.

Female students scored significantly higher than males (t = −2.21, p < 0.05), consistent with prior research (34, 35), and the high smartphone-addiction group scored higher than the low group (t = −3.24, p < 0.01; **Table 5**).Because smartphone addiction is a correlate rather than a criterion of NSSI, this comparison serves as supplementary known-groups evidence.

**Table 5:** Comparison of ANSSI-SF scores across different subject groups.

Sample	Group	Non-suicidal self-injury (Mean ± SD)	t
Sample 1(n=1090)	Male(n=580)	1.33 ± 5.45	-2.21*
	Female(n=510)	2.34 ± 9.28	
Sample 2(n=218)	Low-score group for mobile phone addiction(n=109)	0.14 ± 0.62	-3.24**
	High-score group for mobile phone addiction(n=109)	2.18 ± 7.01	

*p < 0.05, **p < 0.01.

To evaluate screening utility, a ROC analysis was conducted using the full ASHS as the reference for NSSI status (non-zero score; 11).The ANSSI-SF total score showed acceptable discrimination (AUC = 0.720, 95% CI [0.684, 0.757]); at the Youden-index-optimal cutoff of ≥ 1, sensitivity was 0.459 (95% CI [0.388, 0.531]) and specificity was 0.980 (95% CI [0.962, 0.989]), with κ = 0.513 (**Table 6**).

**Table 6:** Screening accuracy of the ANSSI-SF against the full ASHS.

Metric	Estimate	95% CI
Area under the curve (AUC)	0.720	0.684–0.757
Optimal cutoff (total score)	1	—
Sensitivity	0.459	0.388–0.531
Specificity	0.980	0.962–0.989
Youden index	0.439	—
Agreement with full ASHS at cutoff (Cohen’s κ)	0.513	—
Correlation with full ASHS	0.889***	—

ROC, receiver operating characteristic; AUC, area under the curve; ASHS, Adolescent Self-Harm Questionnaire. ***p < 0.001.

### 3.5 Cross-validation based on genetic algorithms

The GA cross-validation (25 independent runs) is illustrated in **Figure 1**: the minimum cost decreased rapidly and stabilized after about 20 generations, the number of selected items stabilized at six, and the average explained variance (R²) stabilized at approximately 0.90. In Stage 1, the six items most predictive of the original ASHS total score (items 5, 6, 7, 9, 10, 12) were concentrated in a few categories (scratching, hitting, piercing), leaving categories such as cutting, tattooing/marking, burning, and other forms unrepresented; this confirmed that an unconstrained GA over-represents the most frequent behaviors while neglecting other categories. To ensure that the simplified scale comprehensively reflects NSSI behavior, these six items were temporarily removed from the item pool, and a second GA with identical parameters was run on the remaining 13 items, yielding items 1, 2, 4, 11, 18, 19.The combined 12-item set fully covered the seven behavioral categories defined *a priori*: cutting (1), hitting (7, 9), scratching (5, 10, 12), piercing (6, 11), burning (18), tattooing/marking (4), and other (19). Items within the same category were highly correlated (hitting: r = 0.76; scratching: r = 0.76–0.81; piercing: r = 0.84; all p < 0.001), indicating substantial content overlap among same-category items. The only divergence concerned item 2, a marginal behavior with high statistical overlap: its correlation with item 1 was 0.68, and although its action mechanism differs from typical cutting, the two behaviors showed high co-occurrence and intrinsic association in adolescent NSSI patterns. On the basis of this statistical evidence and the common comorbidity patterns observed in clinical practice, item 2 was subsumed under the cutting category in the subsequent construction of the short form—a data-supported decision that also ensured the clarity of the simplified scale’s structure.

Relationship to the final scale. The 12-item GA-derived set is cross-validation evidence, not an alternative short form: it shows that full content coverage requires all seven categories and that one item per category suffices. The final seven-item ANSSI-SF (**Table 1**) is therefore the most parsimonious solution achieving full coverage: within each category, the final item was formed by merging the GA-selected items of that category (cutting: items 1 and 2; hitting: items 7 and 9; scratching: items 5, 10, and 12; piercing: items 6 and 11) or, where a single item was selected, by retaining it verbatim (burning: item 18; tattooing/marking: item 4; other: item 19).Overall, the GA cross-validation supports the content breadth and statistical efficiency of the final scale.

## 4 Discussion

Drawing on the Chinese-language ASHS (12), the present study developed and validated the Adolescent Non-Suicidal Self-Injury Scale–Short Form (ANSSI-SF), a seven-item short form whose psychometric properties were rigorously evaluated. The results indicate that the short form demonstrates good reliability, with both internal consistency and split-half reliability reaching satisfactory levels.

Regarding construct validity, findings from exploratory and confirmatory factor analyses jointly support a stable unidimensional structure, confirming the scale’s ability to effectively capture the core characteristics of adolescent non-suicidal self-injury behaviors. Although Item 6 showed relatively lower statistics, it was retained because it represents a distinct NSSI type (object insertion) not covered by other items, and its statistics remained above conventional thresholds. In terms of criterion-related validity, the scale exhibited moderate to high positive correlations with multiple relevant measures, including the Adolescent Self-Harm Questionnaire, depression (PHQ-9), anxiety (GAD-7), experiential avoidance (AAQ-II), sleep quality (PSQI), and mindfulness attention (CAMM), further substantiating its external validity. Given that higher CAMM scores in this study indicate lower mindfulness, the positive CAMM–NSSI correlation is consistent with the theoretically expected inverse association between mindfulness and self-injury.

In analyses of group differences, the scale also demonstrated good discriminant validity. For example, when participants were grouped by gender and smartphone addiction severity, female adolescents scored higher than males on self-injurious behaviors, with statistically significant differences in non-suicidal self-injury tendencies. These findings are consistent with previous research (34, 36), indicating that the scale can effectively distinguish subgroups with differing risk profiles. Such results have important implications for developing targeted prevention strategies (21, 35) and highlight the need to consider the complex interplay between gender-specific factors and emerging behavioral issues in the digital age, alongside traditional self-harm behaviors.

The core contribution of this study lies in its systematic simplification strategy, which combines theoretical and data-driven approaches. Beyond applying a theoretical framework and item selection informed by factor analysis, this study incorporated genetic algorithms for cross-validation. The genetic algorithm results not only confirmed the statistical efficiency of the simplified items but also demonstrated that the two-stage selection process yielded a set of items covering all predefined behavioral categories comprehensively, ensuring that the simplified scale retains content breadth and representativeness while achieving greater efficiency. To our knowledge, this is the first application of a genetic algorithm to abbreviate the ASHS or any Chinese adolescent NSSI instrument. Overall, the cross-validation supports that the final simplified scale possesses a robust theoretical foundation and meets criteria for statistical optimality and practical applicability.

Overall, the ANSSI-SF demonstrates robust psychometric properties, meets established measurement standards, and is well-suited for assessing non-suicidal self-injury behaviors among adolescents in China, providing a more efficient and contextually appropriate assessment tool for both research and clinical practice. Compared with the original scale, the brief scale developed in this study offers several advantages. First, the short form consists of only seven items, reducing the original scale by 12 items; despite this substantial reduction, it effectively captures core self-injurious behaviors while significantly shortening administration time, thereby minimizing participant fatigue or emotional distress that can arise from lengthy assessments. This makes the scale particularly suitable for adolescents who are emotionally sensitive or have limited cognitive resources, improving both participation rates and data quality in clinical screenings and school-based psychological surveys. Second, the scale’s brevity and ease of administration facilitate large-scale screening and dynamic monitoring, allowing flexible integration into various mental health assessment batteries or online survey platforms across schools, communities, and clinical outpatient clinics. As such, the ANSSI-SF provides a practical tool for regional monitoring, routine surveillance, and early intervention of non-suicidal self-injury behaviors among adolescents, demonstrating strong practical utility and substantial potential for widespread adoption.

### 4.1 Limitations and future research

This study has several limitations. First, convenience sampling from two schools in a single region limits generalizability; stratified or cluster sampling across diverse settings is needed. Second, the cross-sectional design precludes predictive-validity assessment. Third, test–retest reliability, measurement invariance (gender, age), and differential item functioning (DIF) were not examined. Fourth, no independent gold-standard NSSI instrument (e.g., ISAS or FASM) was administered; the strong correlation with the full ASHS partly reflects shared item content. Fifth, NSSI scores are highly skewed, which may affect correlational analyses. Sixth, the GA-based procedure involves researcher choices (e.g., the two-stage design, parameters) that warrant replication in independent samples. Future research should also evaluate screening performance in clinical samples and sensitivity to change over time.

## 5 Conclusion

The Adolescent Non-Suicidal Self-Injury Scale–Short Form (ANSSI-SF) demonstrates robust psychometric properties, including satisfactory reliability, construct validity, consistency with the parent ASHS, and known-groups validity, while substantially reducing administration burden. It provides a reliable, brief, locally developed instrument for the rapid identification, systematic evaluation, and early screening of non-suicidal self-injury among Chinese adolescents. These characteristics confer both theoretical and practical value and support the scale’s potential application in research, school-based mental-health screening, and large epidemiological surveys.

## Data Availability

The raw data supporting the conclusions of this article will be made available by the authors, without undue reservation.
